# Genotypic and Phenotypic Characterization of Highly Alkaline-Resistant *Carnobacterium maltaromaticum* V-Type ATPase from the Dairy Product Based on Comparative Genomics

**DOI:** 10.3390/microorganisms9061233

**Published:** 2021-06-06

**Authors:** HyeongJin Roh, Do-Hyung Kim

**Affiliations:** Department of Aquatic Life Medicine, College of Fisheries Science, Pukyong National University, Busan 48513, Korea; hjroh@pukyong.ac.kr

**Keywords:** lactic acid bacteria, genomics, bioinformatics, V-type ATPase, alkaline resistance, food quality

## Abstract

Although *Carnobacterium maltaromaticum* derived from dairy products has been used as a lactic acid bacterium industrially, several studies have reported potential pathogenicity and disease outbreaks. Because strains derived from diseased fish and dairy products are considered potentially virulent and beneficial, respectively, their genotypic and phenotypic characteristics have attracted considerable attention. A genome-wide comparison of 30 genome sequences (13, 3, and 14 strains from diseased aquatic animals, dairy products, and processed food, respectively) was carried out. Additionally, one dairy and two nondairy strains were incubated in nutrient-rich (diluted liquid media) and nutrient-deficient environments (PBS) at pH 10 to compare their alkaline resistance in accordance with different nutritional environments by measuring their optical density and viable bacterial cell counts. Interestingly, only dairy strains carried 11 shared accessory genes, and 8 genes were strongly involved in the V-type ATPase gene cluster. Given that V-type ATPase contributes to resistance to alkaline pH and salts using proton motive force generated via sodium translocation across the membrane, *C. maltaromaticum* with a V-type ATPase might use nutrients in food under high pH. Indeed, the dairy strain carrying the V-type ATPase exhibited the highest alkaline resistance only in the nutrient-rich environment with significant upregulation of V-type ATPase expression. These results suggest that the gene cluster of V-type ATPase and increased alkaline resistance of dairy strains facilitate adaptation in the long-term ripening of alkaline dairy products.

## 1. Introduction

Lactic acid bacteria (LAB) are among the most well-known bioresources widely used industrially. For example, they are used in the ripening of dairy products and in probiotics, and their role and importance in ripening soft cheeses are well established [1,2,3]. The appropriate composition of microbiota during cheese ripening can enhance food texture, flavor, and safety, and greatly influence cheese characteristics such as odor, color, appearance, hardness, and pH [1,4,5,6]. In general, because most LAB participate in acidic microbial processes via lactate fermentation during the first phase of the ripening of soft cheese, the pH of dairy products is decreased [7]. Subsequently, however, ammonia-producing microorganisms (e.g., *Penicillium caseicolum* and *Geotrichum candidum*) metabolize lactic acid and produce ammonia (NH_3_), which creates an alkaline environment in the ripening of dairy products [5,8]. These phenomena directly affect the switch in the composition of microbiota from aciduric to nonaciduric bacteria [1,5,9]. Currently, nonstarter LAB (NSLAB), which are predominantly isolated from the ripening of cheese at low temperature and alkaline conditions, are useful in preventing spoilage and inhibiting pathogenic bacteria such as Listeria [10].

*Carnobacterium maltaromaticum* (formerly *C. piscicola*) has been known as nonaciduric and psychrotrophic NSLAB [1,10]. *C. maltaromaticum* is predominantly found in cheeses during the ripening phase under increased pH and cold storage and may have a positive effect on the preservation of cheeses and the inhibition of Listeria in dairy products by secreting bacteriocins [1,10,11]. Because *C. maltaromaticum* has been isolated from a wide range of environments such as fish, food, sea, and dairy products, its genetic diversity and usefulness are not confined to dairy products [12,13,14]. However, in recent years, some studies [15,16] have warned that specific strains of *C. maltaromaticum* derived from diseased fish could trigger mortality in fish from severe histopathological changes such as ulcers, inflammation, necrosis, and massive hemorrhage in the skin, head kidney, and swim bladder. Nonetheless, because the strains obtained from dairy products are still regarded as beneficial LAB, the importance of the genetic differences between useful dairy *C. maltaromaticum* and other nondairy strains has received great attention [12,17]. When rainbow trout were challenged intraperitoneally with approximately 10^8^ CFU of *C. maltaromaticum* isolated from different sources, only strains derived from diseased fish, but not dairy sources, induced mortality by 60 to 80% [15]. Thus, their genotypic and phenotypic characteristics differed according to the isolation source. Hence, it is necessary to elucidate the phenotypic and genotypic differences between the strains. Therefore, the purpose of this study was to understand the genotypic and phenotypic differences based on comparative genomic analysis. The results will significantly contribute to our understanding of *C. maltaromaticum* as a useful bacterial resource as well as a potential pathogen.

## 2. Materials and Methods

### 2.1. Comparative Genomic Analysis

Whole-genome sequences of 30 *C. maltaromaticum* strains, including three strains derived from dairy products and 13 and 14 strains from diseased fish and processed food, were collected from GenBank. The CDS of all strains were predicted using the RAST server [18,19,20]. Based on profiling the predicted gene counts and existence, a three-dimensional PCA plot and a dendrogram with the complete method were drawn in R (Ver. 3.6.2) using the rgl package [21]. The predicted CDS were functionally annotated using the KEGG database with the condition of Taxonomy group = Prokaryotes and KEGG database = species of prokaryotes, and the accessory ko_id, ko_terms or pathway were selected only in dairy strains.

### 2.2. Existence of V-Type ATPase Subunit A (ATPVA) and V-type ATPase Subunit I (ATPVI) Genes in C. maltaromaticum

*C. maltaromaticum* 18ISCm strain was isolated from diseased trout [15], and *C. maltaromaticum* ATCC 35586 and DSM 20342 were derived from the Korean Collection for Type Cultures (KCTC). All *C. maltaromaticum* strains were cultured on Tryptone Soya Agar (TSA; Oxoid, Hampshire, UK) at 28 °C for 24 to 48 h. Genomic DNA from three strains was extracted using the AccuPrep^®^ Genomic DNA Extraction Kit (Bioneer, Daejeon, Korea), and conventional PCR was used to detect V-type ATPase with the primer sets targeting subunit A (ATPVA; F: 5’-CCC GTC ACG ACA ACT GGT AA-3’, R: 5’-ACG TTC TCC ACA CCC AAC AT-3’) and subunit I (ATPVI; F: 5’- GGG ATG ATG GTT GCG GAT GT-3’, R: 5’-CAC CCG CTA TTC CTA GTG CC-3’). In this study, primers were designed using Primer 3 plus software [22]. Polymerase chain reaction (PCR) was performed under the following conditions: 1 cycle of 95 °C for 5 min; 30 cycles of 95 °C for 30 s, 58 °C for 30 s, and 72 °C for 30 s; and 1 cycle of 72 °C for 5 min using AccuPower^®^ PCR Premix (Bioneer, Daejeon, Korea). Gel electrophoresis was performed with 1% agarose gel. DNA amplicon and size were observed using Gel Doc^TM^ 2000 (Bio-Rad, CA, USA). DNA amplicon was also used for sequencing using a Sanger sequencer (Applied Biosystems 3730XL DNA Analyzer, Applied Biosystems, MA, USA). Additionally, in silico PCR was performed for all 30 *C. maltaromaticum* strains using in silico PCR amplification version 1.0 (Joseba Bikandi; http://insilico.ehu.es/mini_tools/PCR/) (Accessing date: 24 December 2020) and predicted the sensitivity of primers for different isolated sources of *C. maltaromaticum*.

### 2.3. Determination of Alkaline Resistance of C. maltaromaticum

The pH of 1/10 TSB medium (composed of 10% TSB and 90% PBS) and PBS was adjusted to 7 and 10 using HCl and NaOH, respectively. Two different experiments (Exp. 1 and Exp. 2) were carried out to determine their alkaline resistance. In Exp. 1, strains 18ISCm, ATCC35586, and DSM20342 of *C. maltaromaticum* were suspended in 8 mL of PBS (pH 7 and pH 10) and 1/10 TSB (pH 7 and pH 10), respectively. Triplicate bacterial cultures with an optical density (OD) of 0.35–0.45 at 630 nm measured with a Sunrise^TM^ spectrophotometer (TECAN, Männedorf, Switzerland) were incubated at 28 °C under slight shaking at 150 rpm. The OD at 630 nm was then periodically measured at 8, 24, 48, and 96 h after inoculation (hpi). The relative OD value at 630 nm was calculated using the formula below.

Relative OD value (%) = (Each time of OD value at 630 nm/Initial OD value at 630 nm) × 100

In Exp. 2, approximately 10^5^ CFU mL^−1^ of each strain was suspended in PBS and 1/10 TSB at pH 7 and 10. Bacterial counts were determined using a method described in a previous study with slight modifications [23]. Briefly, 5 mL of bacterial suspension in triplicate were prepared and incubated under the same conditions as in Exp 1. At 24 and 48 hpi, 100 μL of 10-fold serial dilutions (10^0^, 10^−1^, and 10^−2^) were spread onto TSA and incubated at 28 °C. Bacterial colonies were counted after incubation for up to 3 days.

### 2.4. Expression of V-Type ATPase Gene of C. maltaromaticum under Alkaline Environment

Each strain (18ISCm, ATCC35586, and DSM20342) was suspended in 8 mL of PBS at pH 7, PBS at pH 10, and 1/10 TSB at pH 7 and pH 10. All bacterial cultures were suspended to attain an OD value of 0.4–0.5 at 630 nm. They were incubated at 28 °C with shaking (150 rpm). At 8, 24, and 48 hpi, 1 mL was obtained, and the total RNA was extracted using TRIzol as previously described [24]. Total RNA (100 ng) was mixed with 2 μL of 50 A_260_ units of random hexamer (Primer random p[dN]_6_, Roche, USA) and DEPC-treated water to obtain a total volume of 10 μL. The mixture was used to synthesize cDNA using an M-MLV Reverse Transcriptase kit (Bioneer, Daejeon, Korea). Most procedures followed the methods described previously [24]. qPCR was performed using an Exicycler^TM^ 96 Real-Time Quantitative Thermal Block (Bioneer, Daejeon, Korea) after 25 μL of AccuPower^®^ 2X Greenstar qPCR Master mix (Bioneer, Daejeon, Korea) was mixed with 2 μL of 16S rRNA (928F-Firm: 5’-TGA AAC TYA AAG GAA TTG ACG-3’, 1040FrimR: 5’-ACC ATG CAC CAC CTG TC-3’) [25] or qATPVA primer set (qATPVA_F: 5’-ATG CAA AAA TGG CCC GTT CG-3’, qATPVA_R: 5’-TCT TTG CCC TGT TGT CAT CG-3’), 5 μL of cDNA, and 16 μL DEPC. The qPCR conditions for the ATPVA gene were: 95 °C for 5 min, followed by 95 °C for 15 s and 61 °C for 20 s. The qPCR conditions for 16S rRNA were: 95 °C for 5 min, followed by 40 cycles of 95 °C for 15 s, 61.5 °C for 15 s, and 72 °C for 20 s [25]. The expression level of ATPVA was normalized against that of 16S rRNA and calculated with 2^−^^△△^^Ct^ [26].

### 2.5. Statistical Analysis

All results are expressed as the mean ± standard deviation (SD). Data were subjected to one-way analysis of variance (ANOVA) based on Duncan’s multiple range test using SPSS version 16.0 (IBM, NY, USA). Significant differences among groups are indicated by different letters.

## 3. Results and Discussion

**Figure 1:**
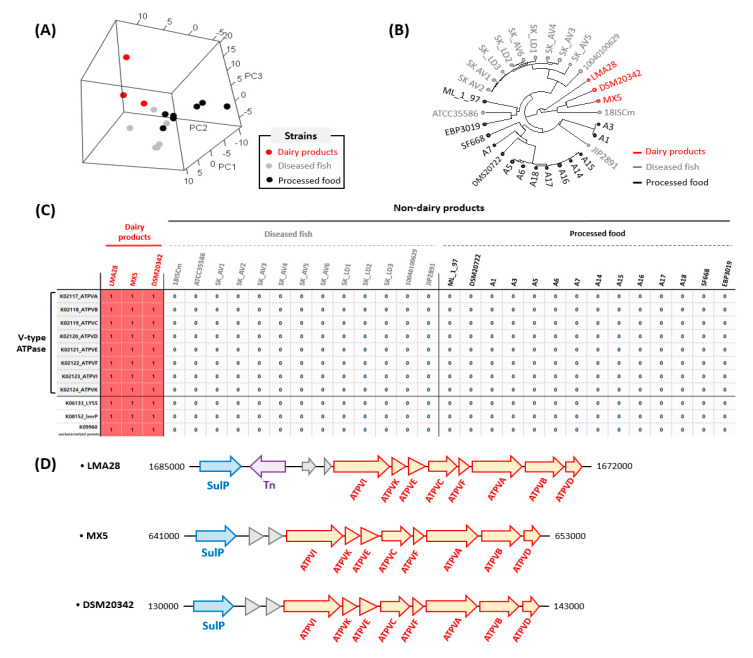
Three-dimensional principal component analysis (PCA) of strains derived from dairy products, diseased fish, and processed food based on the profiling of KEGG Ontology (ko) genes (**A**). Clustering of 30 strains of *Carnobacterium maltaromaticum* isolated under different conditions (**B**). The accessory genes and ko_id that shared only dairy strains among 30 strains of *C. maltaromaticum* (**C**). The serial array of sulfate permease (SulP), transposase (Tn), and the components of V-type ATPase (ATPVA, ATPVB, ATPVC, ATPVD, ATPVE, ATPVF, ATPVI, and ATPVK) in the dairy strains of chromosomal DNA (**D**).

The maximum and the minimum number of protein-coding sequences (CDS) among 30 strains of *C. maltaromaticum* were 3952 and 3226 in 18ISCm and ML-1-97, and an average of 3386 CDS was found in *C. maltaromaticum* (Table S1). On average, 53% of the CDS were annotated in the Kyoto Encyclopedia of Genes and Genomes (KEGG) database (more detailed information is available in Table S2). Principal component analysis and a dendrogram based on the number of KEGG ontology (KO) genes in 30 *C. maltaromaticum* strains showed that dairy strains had similar gene-harboring tendencies, unlike the strains from diseased fish and processed food (Figure 1A,B). Based on comparative genomic analysis, only 11 genes were related to dairy-derived strains (LMA28, XM5, and DSM20342), as shown in Figure 1. These genes were associated with V-type ATPase, LYS5 (4’-phosphopantetheinyl transferase), ImrP (MFS transporter), and an uncharacterized protein. Nevertheless, all *C. maltaromaticum* strains used in this study carried an F-type ATPase (data not shown), and the gene cluster for V-type ATPase that localized in the chromosome was found only in the dairy strains by KEGG pathway analysis. All components of V-type ATPase (ATPVA, ATPVB, ATPVC, ATPVD, ATPVE, ATPVF, and ATPVI, and ATPVK) were serially localized in the chromosomal DNA in the dairy strains next to a sulfate permease (SulP) gene (Figure 1D).

**Figure 2:**
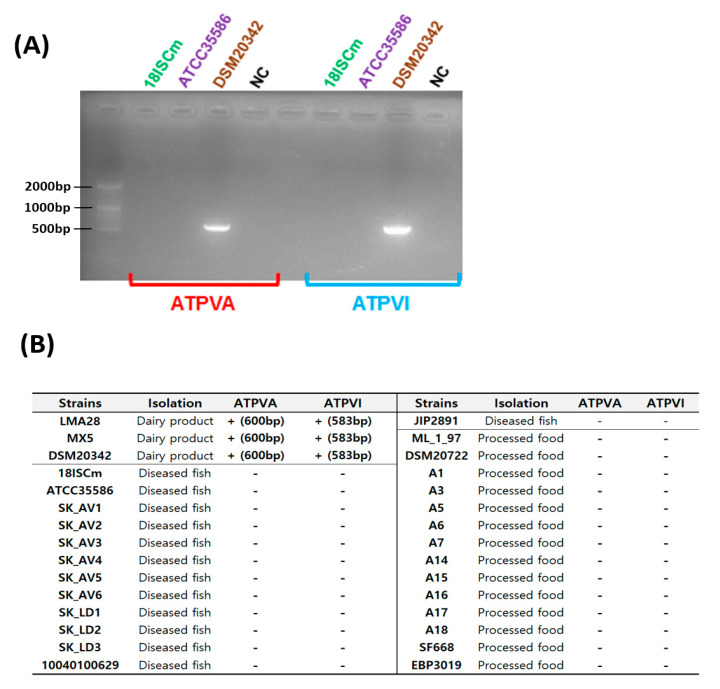
PCR products (ATPVA- and ATPVI-specific primers) using 18ISCm, ATCC35586, and DSM20342 genomic DNA (**A**). The results of in silico PCR for all 30 strains. The results of in silico PCR for all 30 strains isolated from dairy products, diseased fish, and processed food. + denotes positive amplification and the expected amplification size is indicated in the parentheses. - denotes no amplification in in silico PCR (**B**).

The genetic markers differentiating the dairy and nondairy strains using the gene cluster of V-type ATPase, ATPVA, and ATPVI were selected as the target genes for designing specific primers. A thick band in the range of 500–600 bp was detected only in the dairy strain (DSM20342), and the sequence of the DNA amplicon was confirmed using a Sanger sequencing method (Figure 2A). Likewise, only the dairy strains showed a positive response without any false-negative amplification in other sources based on in silico PCR targeting ATPVA and ATPVI among all of the uploaded genome sequences (Figure 2B). This result indicated that only a few *C. maltaromaticum* strains contained V-type ATPase, and all of them originated in dairy products. Vacuolar-type (V-type) ATPase, which pumps sodium via ATP hydrolysis, has been detected only in a few bacteria, including LAB (e.g., *Enterococcus hirae*) and archaea [27,28,29,30]. In general, V-type ATPase plays a crucial role in maintaining physiological homeostasis under extreme alkaline pH and salt concentrations via an active transport system [31,32]. Krulwich et al. [31] reported that the transmembrane electrical potential (∆ψ) was very important in resisting acidic and alkaline environments. In an acidic environment, bacteria try to maintain a positive ∆ψ (intracellular positive charge) to facilitate the discharge of intracellular protons. However, bacteria in an alkaline environment maintain a negative ∆ψ (intracellular negative charge) for attracting external protons via monovalent cation/hydrogen antiporters. In the alkaline environment, V-type ATPase plays a crucial role by pumping Na^+^ from the intracellular to the external area, which greatly contributes to maintaining a negative ∆ψ. Accordingly, the electrical gradients are the driving force moving protons to the intracellular area through the monovalent/hydrogen antiporter found in all *C. maltaromaticum* (Table S2). However, because V-type ATPase, which is a membrane protein, has been known to pump sodium or proton ions [33,34], the possibility that H^+^ is directly transported by V-type ATPase remains.

**Figure 3:**
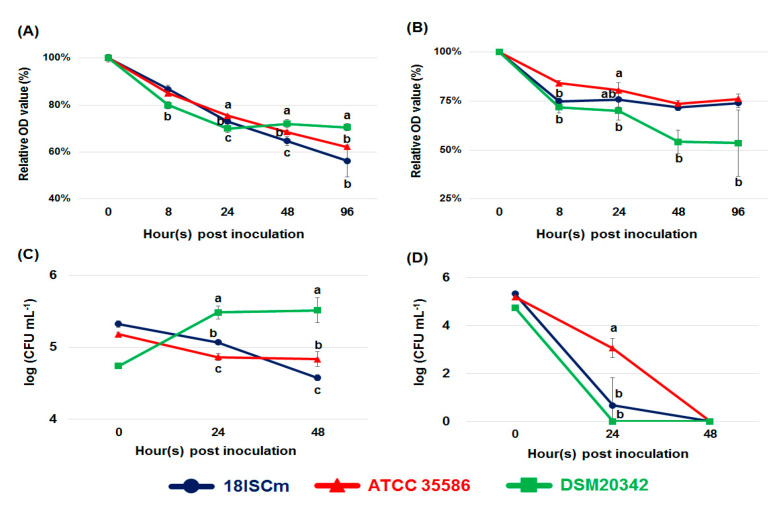
Relative OD value at 8, 24, 48, and 96 hpi in 1/10 TSB (pH 10; (**A**)) and pH 10 PBS (pH 10; (**B**)), respectively. Viable bacterial count at 24 and 48 hpi in 1/10 TSB (pH 10: (**C**)) and pH 10 PBS (pH 10; (**D**)), respectively. Different letters indicate statistically significant differences determined by Duncan’s multiple range test under the same sampling time points (*p* < 0.05).

To verify the phenotypic characteristics of V-type ATPase, we evaluated the resistance of strains carrying the enzyme in a highly alkaline environment under both nutrient-rich and nutrient-deficient culture conditions. The relative OD values of all bacterial cultures in PBS (pH10, nutrient-deficient medium) and 1/10 TSB (nutrient-rich medium) continuously decreased over time in Exp. 1. The relative OD value of DSM 20342 was higher than that of 18ISCm or ATCC 35586 in 1/10 TSB (pH 10) based on an OD of higher than 70% at 48–96 hpi. In contrast, the relative OD value of the nondairy strains (18ISCm and ATCC 35586) in 1/10 TSB at pH 10 was around 60% or less (Figure 3A). However, these patterns were completely reversed in the nutrient-deficient medium (PBS at pH 10). The relative OD value of DSM20342 in PBS (pH 10) was approximately 50% at 48 and 96 hpi, which was significantly lower than that of the nondairy strains at relative OD values higher than 70% (Figure 3B). A similar pattern was observed in Exp. 2. Although 10-fold higher viability of the DSM20342 strain was observed in 1/10 TSB (pH 10) at 24 and 48 hpi compared to the initial concentration, 18ISCm and ATCC 35586 did not grow under similar conditions, and instead, the viable bacterial count decreased compared to that of the initial inoculum (Figure 3C). However, DSM20342 showed no significant differences or comparatively weaker alkaline resistance in PBS at pH 10 compared to other strains (18ISCm and ATCC 35586), with no surviving DSM20342 strain at 24 hpi (Figure 3D). Figure 3A shows that the OD value of DSM20342 decreased, whereas Figure 3C shows the number of its CFU increased. This was thought to be the result of different initial concentrations. In Exp. 1, the initial OD values were adjusted between 0.35 and 0.45, in which the concentrations at the time were approximately 10^8^–10^9^ CFU mL^−1^. However, an initial concentration of 10^4^–10^5^ CFU mL^−1^ was used in Exp. 2. Given that we used 1/10 TSB media because all powders were not dissolved at pH10, the *C. maltaromaticum* cultured for Exp. 1. was in poor-nutrient environments compared to Exp. 2.

**Figure 4:**
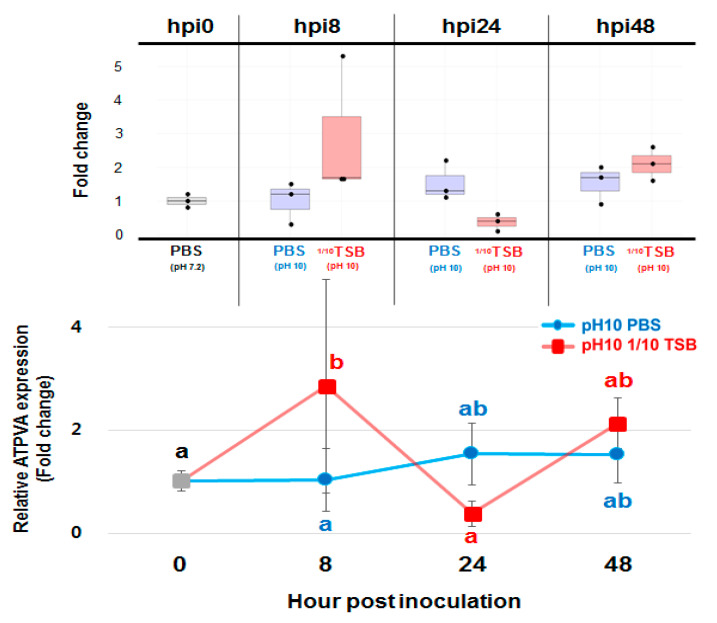
Relative V-type ATPase Subunit A (ATPVA) gene expression in DSM20342 strain under PBS and 1/10 TSB at pH 10. The dots in the box plot indicate the fold change of each sample compared to hpi0. Different letters indicate statistically significant differences determined via Duncan’s multiple range test among all groups (*p* < 0.05).

These differences would result in different growth patterns even though they were incubated in the same media. To investigate the effect of V-type ATPase activation under a highly alkaline environment with or without nutrients, ATPVA expression, one of the major V-type ATPases, was analyzed in both pH 10 PBS and 1/10 TSB at 8, 24, and 48 hpi using the DSM20342 strain. DSM20342 suspended in pH 7.2 PBS was used as the control. Interestingly, the ATPVA expression was more than two-fold higher only in 1/10 TSB at 8 hpi, but no significant difference was observed in the pH 10 PBS group (Figure 4). This result was strongly consistent with bacterial survival in pH 10 PBS and 1/10 TSB, and the selective resistance of DSM20342 in the nutrient-rich environment was probably mediated by the activation of V-type ATPase in the dairy strain.

**Figure 5:**
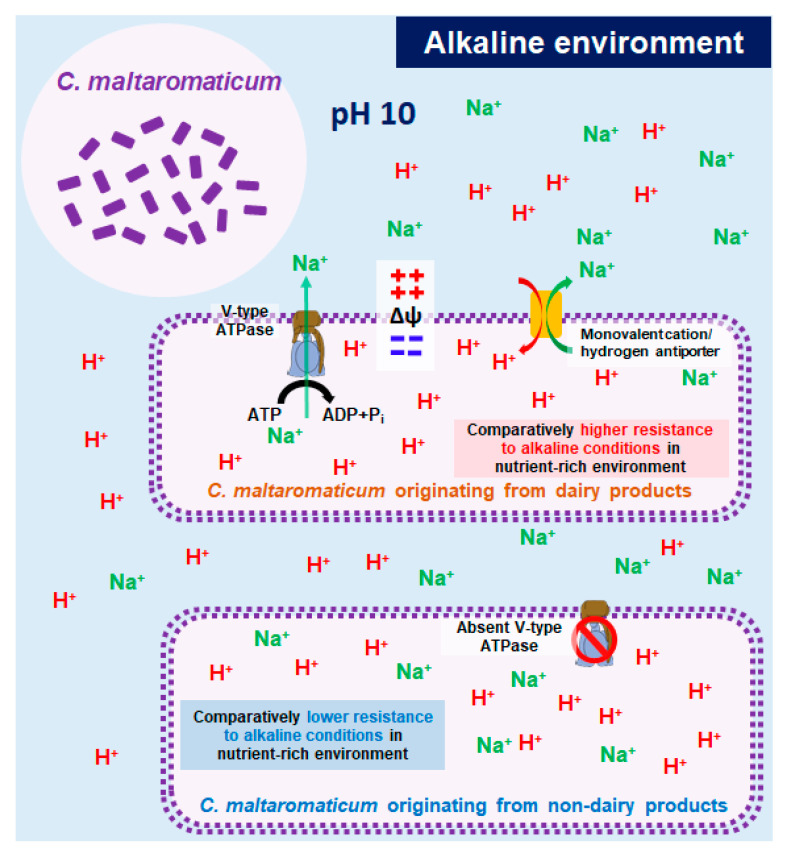
Schematic diagram about the mode of action for V-type ATPase, which only exists in dairy *C. maltaromaticum* under a highly alkaline environment. ∆ψ indicated the electrical potential in intra- and extra-cellular areas.

According to Zhang et al. [33], V-type ATPase in *Enterococcus faecium* was also upregulated during bile salt exposure, and proton or sodium gradients were generated by V-type ATPase under bile salt stress. In prokaryotes, the proton motive force was increased when ATPase was activated, which induced high tolerance under a harsh external environment in some Gram-positive bacteria such as *Lactobacillus plantarum* and *B. longum* [35,36]. Particularly, because protons can neutralize alkaline substances, the activation of V-type ATPase in *C. maltaromaticum* would be very beneficial to surviving in highly alkaline environments. In general, *C. maltaromaticum* exhibits high-alkaline resistance compared to other LAB [37]. This characteristic has been used to develop a selective *C. maltaromaticum* (CM) medium composed of several antibiotics (vancomycin; 3.5 mg L^−1^, gentamycin; 5.0 mg L^−1^, and nalidixic acid; 20 mg L^−1^) with high pH (pH 8.8), to isolate *C. maltaromaticum* from many dairy sources [37,38]. Afzal et al. [38] also reported that some strains of *C. maltaromaticum* could grow in highly alkaline conditions up to pH 9.6. In this study, we found that the activation of V-type ATPase, which was only shown in dairy-sourced *C. maltaromaticum*, could greatly contribute to higher alkaline resistance (Figure 5). Ripening promotes the eventual formation of ammonia, which changes the acidic environment of ripening cheese to an alkaline condition [5,9]. Mei et al. [9] showed a decline in the pH of ripening cheese below 5.5 after 5 days of ripening, but the environment turned alkaline after 30 to 35 days of ripening. Likewise, Leclercq-Perlat et al. [5] observed a switch from an acidic to an alkaline environment (approximately pH 8) within 10 days of ripening. Bubelová et al. [39] analyzed the ammonia levels during the long-term storage of cheeses and reported that the ammonia content increased steadily at 23 and 40 °C over two years, which implies a constant increase in pH. Given that the long-term storage of cheeses occurs over two years [39], the dairy strains harboring V-type ATPase are adapted to long-term survival and metabolism in the alkaline conditions of dairy product storage. Our results implied that the *C. maltaromaticum* strains exhibited different selectivity in highly alkaline environments because of the existence of V-type ATPase, which could lend important genotypic and phenotypic characteristics to differentiate dairy and nondairy strains. It is common that prokaryots acquire new genes, paralogs of existing genes, and xenologous gene displacement (orthologs from another lineage (xenolog) through horizontal gene transfer events) [40,41]. The long-term persistence of horizontally transferred genes usually confers a selective advantage on the recipient organism [40]. Although this study has a limitation that only three dairy strains were used for comparative genomics, the existence of V-type ATPase in only the dairy *C. maltaromaticum* strains might be the result of evolving an optimal genetic composition to adapt to the alkaline dairy environments.

## 4. Conclusions

This study found that only dairy strains carried V-type ATPase based on comparative genomic analysis of 30 *C. maltaromaticum* strains. V-type ATPase consumes ATP in a nutrient-rich environment, enabling the influx of protons into the cell, which results in the development of resistance to highly alkaline conditions. Taking into account that dairy *C. maltaromaticum* has to survive and stay in alkaline-ripening dairy products for a long time, harboring the V-type ATPase could facilitate their proper adaptation to the environment. We suggest that V-type ATPase could be an important genetic feature used by *C. maltaromaticum* in dairy products to adapt to the alkaline environment resulting in the phenotypic characteristic of high-alkaline resistance in nutrient-rich environments.

## Data Availability

All genomic results used in this study are available in GenBank. The accession numbers are listed in Table S3.

## Supplementary Materials

The following are available online at https://www.mdpi.com/article/10.3390/microorganisms9061233/s1, Table S1: The results of RAST annotation for all strains used in this study, Table S2: The number of different ko_id and terms in each strain, Table S3: GenBank, BioSample, and genome assembly accession numbers used in this study.
